# Medical Opinion and Movement

**Published:** 1908-01-11

**Authors:** 


### Medical Opinion and Movement.

The discovery of the mosquito as a carrier of malarial infection and of a similar connection between the tsetse fly and the trypanosome of sleeping sickness has naturally turned the attention of the profession to other winged insects as possible agents of disease, and the common fly has not escaped suspicion. We have in a previous issue referred to researches which have shown that flies are probably very potent sources of infection. A report has recently been published of the bacteriologist of the Water Bureau of New York City on investigations to determine the influence of river flies in conveying the germs of disease to the people of the city. Flies were collected in traps placed underneath the piers of the city, and examination showed that a "South Street housefly" carried 100,000 bacteria. Flies were thickest at the points where there was most sewage. By tabulations and diagrams it is shown that flies were most prevalent during the hot season, and a record of deaths from intestinal diseases shows that the mortality rose and fell with the rise and fall in prevalence of the flies. The report concludes by asserting that the fly is one of the chief sources of infection, and is responsible for about 600 deaths from typhoid fever, and about 7,000 deaths from other intestinal diseases annually in New York City.

In the present overcrowded condition of the medical curriculum, and with each of the many "—ologists" clamouring for more of the time and attention of the medical student, it is somewhat consoling to find a teacher in one of the branches of study suggesting a curtailment of his particular subject. Yet this is the sum and substance of an admirable address by Dr. Arthur Keith to the students of the London Hospital Medical College. Our modern conception of human anatomy, he tells us, is *to describe*, and in adopting this we have deserted the ideals set up by William Harvey, John Hunter, Munro, and the two Bells, and followed the French school of anatomists. "Up to the end of the eighteenth century there was a strong school of British anatomists, who regarded dissecting as a means for obtaining not a description, but an understanding of the human body." Now structure is divorced from function, and the elaborate modern text-book of anatomy contains an exhaustive description of the *dead* human body. Dr. Keith suggests that the old ideals should be again recognised, and that the anatomical studies of the medical student should be modified and regulated with a view to a knowledge of the human body as a living mechanism. Less attention should be given to a detailed and minute description of the origin and insertion of the muscles, and more to the function of the muscles as a mechanism for movements. Such considerations are of undoubted medical value, while the details of modern descriptive anatomy are so much cumbrous lumber, which, fortunately, the brain knows how to dispose of speedily, when the necessary examinations have been passed.

The vexed question of the relation of syphilis to the so-called parasyphilitic diseases of the nervous system—locomotor ataxia and general paralysis—is carefully considered in a recent article by Dr. F. W. Mott, F.R.S., and the pathological hypothesis which he puts forward to explain the connection is ingenious and suggestive. While admitting that conditions such as alcohol, sexual excess, and severe mental strain may play an important *rôle* in the development of these diseases, he is convinced of the causal connection between them and the syphilitic virus. On the other hand, he discards the view that they are a late manifestation of the poison comparable to other true syphilitic lesions of the late period. He suggests that the syphilitic virus causes the nerve cells to exercise an abnormal metabolic activity in the production of the sick-chain molecules necessary for immunisation against the toxic effects of the poison, and this excessive activity of the nerve cells, together with the strain from the conditions above referred to, leads to an early decay and degeneration of the nerve elements manifested in these parasyphilitic conditions. In this way the uselessness of antisyphilitic remedies is accounted for, and in fact they are generally positively injurious, by lowering the vitality in a system already over-immunised against the syphilitic virus.

In the annual report just issued by the Board of Education for 1906-1907, considerable attention is naturally devoted to the working of the new Act providing for the medical inspection of the children in elementary schools. But in other ways, also, the Board show that they are thoroughly alive to the necessity of providing for the physical development of the body as well as for the training of the mind, and they recognise that in all schools provision should be made for recreative exercises to be carried out daily on the principles underlying definite physical training. This work must be undertaken by the regular staff, and for this purpose it is essential that more attention should be given to physical training in the training colleges. The report points out that it is not sufficient that the students in the colleges obtain the necessary physical exercise for their own health, but they should leave the training college properly equipped with the necessary knowledge to enable them to give the physical exercises to children in the public elementary schools. Physical training must be considered at least as important in the elementary schools as lessons in history and geography, and recognition of this fact must be found in the curricula of the training colleges. These are excellent sentiments, with which the whole medical profession will heartily concur, and we can only hope that they will be speedily embodied in practical measures. The question of physical training is closely connected with that of medical inspection. All children must not be submitted to the same methods of training, the physically feeble or defective must be separated by careful medical inspection, and special exercises devised adapted to their peculiar condition.

